# ApoE in Alzheimer’s disease: pathophysiology and therapeutic strategies

**DOI:** 10.1186/s13024-022-00574-4

**Published:** 2022-11-08

**Authors:** Ana-Caroline Raulin, Sydney V. Doss, Zachary A. Trottier, Tadafumi C. Ikezu, Guojun Bu, Chia-Chen Liu

**Affiliations:** Department of Neuroscience, Mayo Clinic, 4500 San Pablo Road, 32224 Jacksonville, FL USA

## Abstract

Alzheimer’s disease (AD) is the most common cause of dementia worldwide, and its prevalence is rapidly increasing due to extended lifespans. Among the increasing number of genetic risk factors identified, the apolipoprotein E (*APOE*) gene remains the strongest and most prevalent, impacting more than half of all AD cases. While the ε4 allele of the *APOE* gene significantly increases AD risk, the ε2 allele is protective relative to the common ε3 allele. These gene alleles encode three apoE protein isoforms that differ at two amino acid positions. The primary physiological function of apoE is to mediate lipid transport in the brain and periphery; however, additional functions of apoE in diverse biological functions have been recognized. Pathogenically, apoE seeds amyloid-β (Aβ) plaques in the brain with apoE4 driving earlier and more abundant amyloids. ApoE isoforms also have differential effects on multiple Aβ-related or Aβ-independent pathways. The complexity of apoE biology and pathobiology presents challenges to designing effective apoE-targeted therapeutic strategies. This review examines the key pathobiological pathways of apoE and related targeting strategies with a specific focus on the latest technological advances and tools.

## The pathogenesis of Alzheimer disease

Alzheimer’s disease (AD) is the most common cause of dementia, accounting for approximately 60–80% of all dementia cases [1]. Despite the broad prevalence and rising incidence of AD, there are only six FDA-approved drugs currently used to treat symptoms including cholinesterase inhibitors, NMDA receptor antagonists, and other neuromodulatory agents that are presently prescribed to AD patients to ease the cognitive symptoms [2]. The newest FDA-approved drug, aducanumab, is an antibody-based immunotherapy designed to remove amyloid-β (Aβ) plaques. The clinical efficacy of this new drug is under debate limiting its application to broad AD patients [3, 4]. Overall, there is an urgent need to develop effective therapeutics to reverse or robustly attenuate pathological progression of AD and associated neurodegeneration.

At the cellular level, AD is characterized by synaptic dysfunction and neurodegeneration, neuroinflammation, as well as vascular dysfunction [5, 6]. This combination of neuronal loss surrounded by a heightened inflammatory state is thought to give rise to the eventual decline in cognition seen in AD patients. The key neuropathological hallmarks of AD include extracellular Aβ plaques and intracellular neurofibrillary tangles (NFTs) composed of aggregated tau protein [7, 8]. Much of what we have learned about the etiology of AD comes from studies of the familial form of early-onset AD (FAD), which occurs before 65 years of age. This hereditary form of AD is primarily caused by specific mutations in the genes encoding amyloid precursor protein (APP), presenilin 1 (PSEN1), and presenilin 2 (PSEN2), essential for Aβ production, suggesting a critical role of Aβ in disease development [9]. However, more than 95% of AD cases are sporadic or late-onset AD (LOAD), where the etiology is heavily influenced by environmental and genetic risk factors [10]. With considerable genetic heterogeneity in LOAD cases, it has been difficult to pinpoint specific genes or pathways that directly lead to the onset of clinical pathology. However, as the strongest genetic risk factor for LOAD [11], the apolipoprotein E (*APOE*) gene clearly impacts the majority of the pathogenic pathways that contribute to AD. As such, targeting apoE offers a unique opportunity to potentially benefit a greater number of AD patients [12].

### ApoE isoform is a risk determinant for AD and related dementias

There are three predominant *APOE* alleles in humans; the ε2 (*APOE2*), ε3 (*APOE3*), and ε4 (*APOE4*) alleles which confer varying levels of disease risk. *APOE4* is a major genetic risk factor for AD in a gene dose-dependent manner increasing risk by up to 15 fold in homozygotes [13], whereas *APOE2* reduces AD risk by almost half and contributes to longevity [14]. In addition to the relatively common variants, there have been various rare variants of apoE identified, such as apoE3-R136S (apoE3-Christchurch; apoE3-Ch), apoE3-V236E (apoE3-Jacksonville; apoE3-Jac,) and apoE4-R251G [15–19], that are thought to confer some protection against AD pathology. These rare mutations are being investigated in the hope of identifying the molecular mechanisms involved in alleviating disease risks and developing novel therapeutic strategies. Mounting evidence demonstrates that *APOE4* increases the risk of developing AD via a combination of gain of toxic effects and loss of protective functions [20]. While *APOE2* has been shown to offer protection against AD-related pathology [21, 22], the mechanisms involved remain unclear. Intriguingly, Insel, et al. has reported that carrying an *APOE2* in the presence of *APOE4* may offer some protection against Aβ accumulation compared to *APOE3* [23]. However, others have found that the odds ratio of *APOE2/4* individuals is more similar to that of *APOE4* carriers than *APOE2* carriers [21], suggesting that the increased risk associated with *APOE4* is more dominant than the protection offered by *APOE2*. The multitude of pathological pathways by which apoE impacts AD risk and disease progression makes it an ideal therapeutic target for AD [24].

*APOE4* has also been shown to increase the risk for other dementias. For example, *APOE4* carriers are more likely to have increased severity of Lewy body pathology, independently of AD pathology [25–27]. Similarly, *APOE4* was shown to increase the risk of Parkinson’s disease dementia as well as reduce the age of symptom onset [28]. More recently, animal studies have revealed that apoE4 regulates α-synuclein pathology and exacerbates its toxic effects independent of amyloid pathology [29, 30]. Additionally, patients with vascular dementia carrying *APOE4* present with greater cognitive impairment compared to other alleles [31]. Thus, gaining an understanding of the mechanisms of apoE in disease pathogenesis will ultimately shed light on therapeutic strategies for the treatment of AD and related dementias. Here, we summarize relevant biological functions of apoE and potential pathological mechanisms that contribute to disease progression. We will further discuss current therapeutic strategies targeting apoE to improve AD pathology and offer insights on how these strategies could be improved based on recent evidence from novel technologies.

## ***APOE biology***: synthesis, structure, and function

**Fig. 1:**
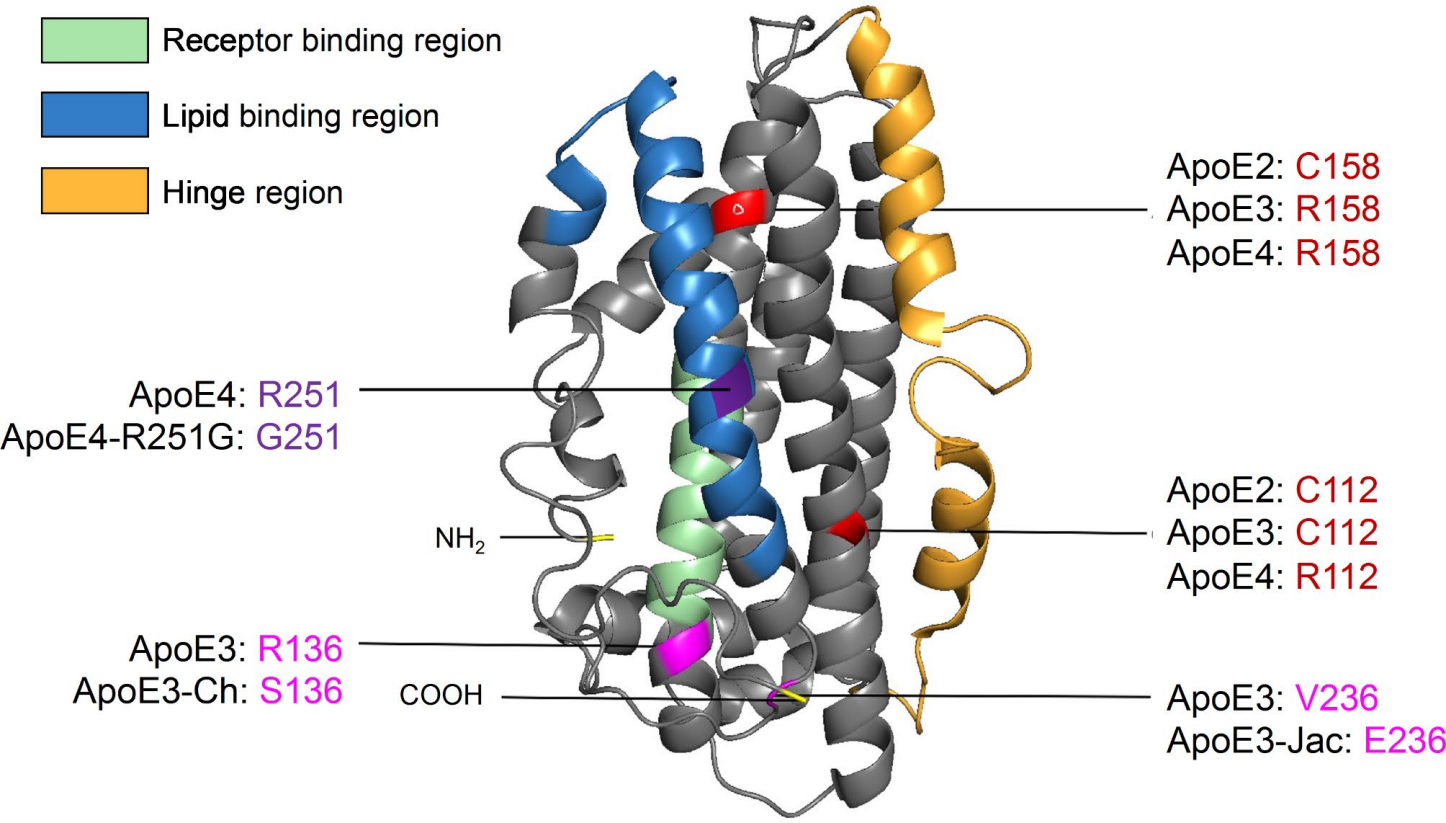
Structural model of apoE highlighting AD-related amino acid variations. ApoE is a 299 amino acid glycoprotein with a molecular weight of 34 kDa (PDB 2L7B). It is formed of two independently folded domains linked by a hinge region: the N-terminal domain (residues 1-167) contains the receptor-binding region while the C-terminal domain (residues 206–299) includes the lipid-binding region. There are three major apoE isoforms that differ at amino acid positions 112 and 158: apoE2 (C112/C158); apoE3 (C112/R158); and apoE4 (R112/R158). Additional rare apoE variants have been identified: apoE3-Christchurch (R136S), apoE3-Jacksonville (apoE3-V236E), and apoE4-R251G

ApoE is a 299 amino acid glycoprotein with a molecular weight of 34 kDa. It primarily functions as a lipid transporter responsible for delivering cholesterol and phospholipids throughout the body. In the periphery, apoE is mainly produced by hepatocytes and macrophages in the liver [32]. While apoE does not cross the blood-brain barrier (BBB), it is also abundantly expressed in the central nervous system (CNS) by astrocytes, activated microglia, vascular mural cells, choroid plexus cells, and to a lesser extent in stressed neurons [33–37]. The three major isoforms of human apoE are distinguished by amino acid positions 112 and 158, which vary between a cystine and an arginine (apoE2: Cys112/Cys158; apoE3: Cys112/Arg158; apoE4: Arg112/Arg158). This single amino acid substitution between apoE4 and apoE3, and apoE3 and apoE2 drastically alters the functionality of apoE, resulting in isoform-specific variations in structure that modulate lipid binding, receptor binding, oligomerization propensity, and stability [38–41] (Fig. 1). Thus, understanding key differences in the apoE structure between isoforms is paramount to understanding its function. Although the precise structure of native apoE is still ambiguous due to the protein’s propensity to aggregate, there is a Nuclear Magnetic Resonance (NMR)-based working model generated from a mutated, monomeric apoE3 [42]. ApoE consists of two main structural domains connected by a hinge region. The N-terminal domain (residues 1-167) contains the receptor binding region [43], while the C-terminal domain (residues 206–299) contains the lipid-binding region [44] (Fig. 1). Lipids are loaded onto apoE via interaction with transmembrane ATP-binding cassette (ABC) transporters such as ABCA1 and ABCG1 [45].

In the brain, apoE plays an important role in transporting cholesterol and other lipids to neurons through binding to cell-surface apoE receptors involved in lipoprotein metabolism, such as low-density lipoprotein receptor (LDLR), and LDLR-related protein 1 (LRP1) [46]. ApoE also interacts with heparan sulfate proteoglycans (HSPG) [47] and binds heparin through two separate sites [48]. Modulation of these apoE receptors has been shown to affect amyloid and tau pathologies [49–52], further supporting the role of apoE in AD pathogenesis. Receptor binding of apoE is dependent on the isoform, lipidation status, and aggregation status of apoE [5, 42]. However, it remains unclear whether downstream receptor-mediated pathways are modulated by apoE isoforms. Understanding these mechanisms will help to fully elucidate isoform-dependent pathways involved in AD pathogenesis and could provide novel therapeutic targeting strategies.

## Physiological function of apoE

ApoE is abundantly expressed in the periphery and in the CNS. However, due to the BBB, apoE in the periphery and in the CNS exist as distinct pools [53]. Therefore, it is critical to consider the independent role that each may play in AD pathogenesis, and the opportunity presented by each for therapeutic intervention. ApoE in the periphery is predominantly produced by the liver [32]. Peripheral apoE maintains lipid homeostasis by participating in the redistribution and metabolism of lipids, such as triglycerides, cholesterol, cholesteryl esters, and phospholipids, through the formation of lipoprotein particles. ApoE isoforms have been shown to be differentially associated with peripheral lipoprotein particles. For instance, apoE4 is mostly found in triglyceride-rich particles like chylomicrons and very low-density lipoproteins (VLDL), whereas apoE2 and apoE3 have preference to the high-density lipoproteins (HDL). Thus, the single Arg to Cys amino-acid substitution at position 112 dictates the differential distribution of apoE isoforms among lipoprotein particles [54]. Single amino acid substitutions in rare apoE isoforms may also modulate their distribution among peripheral lipoproteins. For instance, apoE3-Jac exhibits higher cholesterol efflux capacity compared to apoE3, suggesting that apoE3-Jac may differentially bind lipids and could be differentially distributed among lipoprotein particles compared to the common apoE isoforms. Peripheral apoE is also important for cardiovascular function and immune modulation, both factors that contribute to AD risk [20, 55]. These effects will be discussed in more detail later in this review.

In the CNS, apoE-mediated cholesterol and lipid transport plays a critical role in synapse formation and tissue repair [5]. It also plays a role in neurite outgrowth following injury in an isoform dependent manner with astrocytic apoE3 inducing greater neurite outgrowth than astrocytes secreting apoE4 [56]. Human apoE4-targeted replacement (TR) mice, as well as human *APOE4* carriers, show a reduction in dendritic spine density even in the absence of disease pathology [57]. This is consistent with studies which revealed that apoE4 alters structural reorganization of neurons [58], reduces expression of key synaptic proteins [59], and inhibits glutamatergic signaling which are critical for neuronal plasticity and network maintenance [60].

Emerging evidence shows that the function of apoE is largely cell type-specific [61, 62]. While apoE expression in the brain was first described in astrocytes, it has been found to be drastically up regulated in activated microglia and in stressed neurons under pathological conditions and injury [34, 36, 63, 64]. Recent reports also suggest that primary astrocytes and microglia express and secrete apoE with varying sizes likely due to different post-translational modifications and lipid compositions, which might contribute to cell type-specific functions in response to injury [65]. Other cell types, such as pericytes and oligodendrocytes, have also been reported to express apoE more abundantly after liver X receptor (LXR) stimulation and after injury [66–69]. Thus, exploring the structure, lipidation status, and biochemical properties of apoE isoforms expressed by individual brain cell types will be critical for understanding apoE-related effects in the brain.

## Pathobiological function of apoE

### ApoE and Aβ

It is well established that apoE co-deposits with Aβ in amyloid plaques [70]. The interaction between apoE and pathological Aβ deposition appears to be a central mechanism by which apoE contributes to AD risk. Knocking out endogenous *Apoe* in amyloid model mice alters the morphology of Aβ plaques from compact to diffused [71, 72], suggesting that apoE may play a major role in Aβ fibrilization and amyloid deposition. Importantly, the effect of apoE on amyloid pathology is shown to be isoform-dependent (apoE4 > apoE3 *>* apoE2*).* ApoE4-TR mice show increased Aβ levels in brain tissues and in the CSF compared to apoE3-TR [73, 74]. Additionally, post-mortem studies of human brain tissue have increased Aβ plaque deposition and earlier onset of amyloid pathology in individuals carrying *APOE4* [75–77]. Positron emission tomography (PET) imaging revealed that *APOE4* carriers show earlier deposition of Aβ [78, 79], as well as greater overall deposition and broader cortical distribution [80–82]. Conversely, *APOE2* carriers showed delayed onset of Aβ deposition, less severe pathology, and protected cognitive function [83]. Studies of Aβ kinetics in the presence of apoE suggests that apoE4 stabilizes soluble, cytotoxic, oligomeric Aβ fragments and enhances fibrillogenesis [84]. In addition, apoE4 has been shown to accelerate early seeding of amyloid pathology. For instance, using mouse models in which apoE isoform expression is conditionally induced, astrocytic expression of apoE4 during the seeding stage of Aβ plaque development, but not in the rapid growth period, resulted in a substantial increase in plaque deposition as well as an increased Aβ half-life in the brain [85]. Similar results were found using APP/PS1-21 animal models treated with antisense oligonucleotides (ASOs) against *APOE4*, where inhibition of apoE4 during the seeding stage leads to larger Aβ plaques with reduced plaque-associated neuritic dystrophy [86]. The findings of earlier Aβ deposition [87], greater Aβ oligomerization, and lower CSF and plasma Aβ levels in human *APOE4* carriers [88, 89] strongly support the idea that apoE isoforms differentially regulate Aβ fibril formation via its interaction with Aβ, and that this interaction may represent a potential target for therapeutic intervention at early stages of the disease.

In addition to promoting Aβ plaque formation, apoE is also involved in the clearance of Aβ via various mechanisms, such as receptor-mediated clearance and proteolytic degradation. LRP1 receptor in neurons is shown to mediate Aβ clearance via the uptake of Aβ/apoE complexes [90–92]. Due to the reduced stability of the complex between apoE4 and Aβ [93, 94], this uptake process is impaired in *APOE4* carriers. This clearance impairment is further compounded by alterations in receptor binding and the competition of apoE for Aβ receptor binding sites [95], resulting in dramatically reduced receptor-mediated clearance in the presence of apoE4. In addition, soluble Aβ can be cleared by proteolytic enzymes, such as metalloendopeptidases, plasminogen activators, matrix metalloproteinases, and lysosomal peptidases [96]. It has been shown that apoE promotes Aβ degradation within microglia in an isoform-dependent manner through neprilysin (NEP) and insulin-degrading enzyme (IDE) in the extracellular space. Enhanced expression of lipidated apoE was also shown to stimulate proteolytic Aβ degradation through LXRs and ABCA1 [97]. Given that apoE4 is a less-effective lipid transporter compared to apoE2 and apoE3, the apoE4-mediated proteolytic degradation of Aβ is also compromised [98], leading to reduced Aβ clearance.

### ApoE and tau

Along with Aβ plaque formation, a major hallmark of AD pathology is the presence of NFTs composed of hyperphosphorylated tau aggregates. ApoE4 has been shown to increase tau phosphorylation compared to apoE2 and apoE3 in the presence of Aβ oligomers [99]. Human studies using PET imaging revealed that *APOE4* carriers show an increased tau deposition both in the presence and absence of Aβ plaques [100]. Neuronal apoE4 was shown to promote tau phosphorylation and cell death compared to apoE3 in induced pluripotent stem cell (iPSC) cultures [101]. Additionally, animal models of tauopathies have shown that apoE4 is associated with greater total tau and phospho-tau levels [102], and exacerbates tau-mediated neurodegeneration by modulating microglial activation [103, 104]. A recent study reported that selective deletion of astrocytic apoE4 can reduce tau-related synaptic degeneration, disease-associated gene signatures, and protect against microglial phagocytosis [105]. Moreover, a study using an adeno-associated virus (AAV)-tau delivery approach found that apoE2 may lead to increased tau phosphorylation and aggregation and is associated with the risk of developing primary tauopathy. The increased tau aggregation is potentially due to the formation of tau/apoE complexes, which primarily occur in the presence of non-lipidated apoE2 [106]. A recent GWAS study indicates that apoE2 distinctly regulates protein phosphatase 2 A (PP2A) activity, a major tau phosphatase in the human brain, which could offer protection against AD risk. The authors further suggest that the protective mechanism of *APOE2* might be distinct from the deleterious effect of *APOE4* on AD risk [107]. These results demonstrate the effects of apoE on the pathogenesis of tauopathies, as well as tau-mediated neurotoxicity, and provide supporting evidence that the role of apoE is isoform-dependent.

The isoform-specific interactions between apoE and tau have become of interest not only in AD research, but also for other tauopathies such as frontotemporal dementia (FTD), chronic traumatic encephalopathy (CTE), and corticobasal degeneration (CBD) [103, 106, 108, 109]. For example, human *APOE4* carriers with FTD, a primary tauopathy, are younger in age at the onset of tau pathology, exhibit signs of exacerbated neurodegeneration, and show greater cognitive decline than non-*APOE4* carriers [108, 110]. Together these findings suggest that apoE influences tau pathology, even independently of Aβ pathology, which contributes to disease progression. Thus, understanding the molecular mechanisms of apoE in the context of tauopathy might provide critical information on developing strategies for AD and other tau-related neurodegenerative diseases.

Early research showed that apoE binds to the region of tau believed to be responsible for aggregation into pathogenic NFTs. It has been postulated that apoE might bind tau directly and block its phosphorylation sites. Such interaction was also observed to be isoform-specific, with apoE3 showing much stronger binding affinity to the microtubule-binding region of tau than apoE4 [111]. The reduced affinity of apoE4 binding to tau may increase the availability and likelihood of GSK3-mediated tau hyperphosphorylation, which leads to an increased formation of NFTs [112]. Alternatively, it has been postulated that apoE4 inhibits the Wnt signaling pathway through LRP5/6 receptors by increasing GSK3 activity, leading to increased tau phosphorylation [113]. These potential mechanisms continue to be studied to better understand the contributions of apoE to tau pathogenesis.

### ApoE and neuroinflammation

A growing body of literature now suggests that inflammation plays a key role in neurodegenerative processes and is subject to modulation by apoE. While apoE contributes to AD pathogenesis in a wide range of pathways, recent data suggests that these different pathways may have a common node in neuroinflammation [114]. Microglia, often observed surrounding plaques in post-mortem brain tissue, have been shown to mediate the inflammatory response and phagocytosis of amyloid plaques [115]. Interestingly, studies have demonstrated that apoE-deficient mice show reduced microglial reactivity to plaques [105, 116, 117], suggesting that apoE may be necessary for the microglial response to amyloid aggregation. Emerging studies show that disease-associated microglia (DAM), or microglial neurodegenerative (MGnD) phenotype, exhibit a conserved transcriptional signature across AD mouse models with *Apoe* serving as one of the central regulators [116, 118]. Furthermore, the impact of apoE on microglial function is likely isoform-specific [119, 120]. A recent report demonstrates that apoE3 is more efficient at inducing microglial response to injected Aβ compared to apoE4, and such observation may be mediated by triggering receptor expressed on myeloid cells 2 (TREM2) [121]. Expressed specifically by microglia in the brain, TREM2 is shown to interact with apoE with high affinity and modulate microglial responses [122]. There is also evidence that binding of apoE to TREM2 is dependent on apoE isoform [123] and lipidation status [124], which may account for differences in microglial function between isoforms. Specifically, apoE4 may impair homeostatic microglial functions compared to other isoforms [125] potentially due to its reduced lipidation and reduced affinity to TREM2.

In the peripheral immune system, *APOE* genotype has been shown to modulate the level of C-reactive protein (CRP) in plasma or serum in multiple cohorts [126–128]. CRP is an inflammatory protein primarily produced by hepatocytes in response to injury or inflammation [129]. Interestingly, *APOE4* carriers show lower levels of CRP relative to *APOE3* individuals and *APOE2* carriers [126–128]. CSF proteomics has also shown a reduction of CRP and complement cascade proteins in *APOE4* carriers versus *APOE2* carriers and *APOE3* individuals. Contrary to this trend in genotype, cumulative incidence of AD was shown to markedly increase as levels of serum CRP increase, with the greatest effect in *APOE4* carriers [126]. However, *APOE* but not *CRP* haplotype was associated with life-long cognitive decline in a longitudinal cohort [130], which does not support a causal effect of CRP on cognitive decline. Therefore, *APOE4* carriers may be subjected to aberrant immune responses to pathological development, which can ultimately lead to harmful effects on injury responses and cognitive deficits. Thus, targeting apoE-mediated inflammatory responses may attenuate AD pathologies and neurodegeneration, and is a valid therapeutic approach to be explored.

### ApoE in dysregulation of lipid metabolism

As a major lipid transporter in the brain, apoE has been shown to mediate lipid and cholesterol metabolism in an isoform-dependent manner [131]. In rat primary astrocytes and neurons, recombinant apoE2 was more efficient at promoting the efflux of cholesterol and phosphatidylcholine (PC), followed by apoE3, and then apoE4 [132]. Deficiency in facilitating cholesterol efflux may result in its intracellular accumulation, which can lead to cytotoxicity. For instance, *APOE4* iPSC-derived astrocytes tend to build up more unsaturated fatty acids and cholesterol compared to *APOE3* astrocytes [62, 133], and are more prone to accumulation of esterified cholesterol stored as lipid droplets [134]. In addition to astrocytes, microglia can also accumulate lipid droplets during aging and under disease-associated conditions [135, 136]. Claes et al. have reported that human iPSC-derived xenografted microglia (xMG) transplanted into the brains of an amyloid mouse model accumulate numerous lipid droplets in the vicinity of amyloid plaques, but not in those distant from plaques [136]. In animal studies, no major changes in lipid profiles were reported in the brains of young and middle aged apoE-TR mice [137]. However, apoE2-TR mice were shown to have lower cholesterol levels in the cortex, and higher levels in CSF and plasma compared to apoE3- and apoE4-TR mice [138], suggesting more efficient cholesterol efflux in apoE2-TR mice.

In human observational cohort studies, individuals with different *APOE* genotypes exhibit distinct lipid profiles in the periphery. *APOE2* carriers present with lower total cholesterol, and *APOE4* carriers with higher total cholesterol compared to *APOE3* carriers [115]. However, both *APOE2* and *APOE4* carriers have higher plasma triglycerides compared to *APOE3* carriers [115]. Lipid dyshomeostasis has also been reported in postmortem AD brains [134] and has been shown to be apoE isoform dependent. For instance, demented *APOE4* carriers display decreased levels of several lipid classes, such as phosphatidylethanolamine (PE) and phosphatidic acid (PA) [139]. The association of apoE4 with lower cholesterol transport capacity and increased lipid droplet accumulation may be a major contributing mechanism through which *APOE4* presents a strong risk for AD.

### ApoE in vascular dysfunction and BBB integrity

The contribution of the vasculature to AD pathogenesis is gaining an increased interest as research has shown that vascular cognitive impairment and dementia (VCID) and AD share some converging pathology and etiology, including the involvement of apoE [140]. ApoE4 intensifies cholesterol dysregulation, stimulates inflammation, promotes metabolic dyshomeostasis, and enhances BBB breakdown, which promotes cerebrovascular damage and increases the risk for both VCID and AD [140]. Interestingly, both *APOE2* and *APOE4* have been found to correlate with increased amyloid accumulation in the parenchymal and meningeal cerebrovasculature, classified as cerebral amyloid angiopathy (CAA) [141]. However, *APOE2* was found to reduce Aβ positivity in patients with AD-related cognitive decline but increase Aβ positivity in patients with VCID, potentially due to the presence of vascular amyloid which increases the risk of CAA [142]. The progression of CAA alters Aβ clearance mechanisms and leads to vascular pathological changes, including augmented vascular pulsation and reduced vascular smooth muscle cell coverage [143]. The reduction of Aβ clearance may be further exacerbated by apoE4, which causes premature shrinkage of meningeal lymphatic vessels, resulting in abnormal lymphatic function [144]. Conversely, apoE-enriched HDL particles have been shown to drastically diminish CAA in bioengineered vessels in an in vitro system by reducing vascular Aβ deposition [145]. Further investigation is required to elucidate how apoE-containing HDL particles reduce CAA, and whether these mechanisms are isoform-dependent.

Vascular mural cells (VMCs), including smooth muscle cells and pericytes are responsible for the homeostasis and function of the cerebrovasculature. It was recently demonstrated that VMC-derived apoE4 leads to a reduction of arteriole blood flow and behavioral deficits, likely due to increased astrogliosis of the vasculature [146]. In addition, cognitively normal *APOE4* carriers have reduced retinal capillary density, which could act as a measurable in vivo model of impaired capillary blood flow in the cerebral vasculature [147]. However, it is interesting to note that *APOE4* carriers have a reduced risk of developing glaucoma due to impaired activation of microglia in the retina [148]. Another study shows that *APOE4* carriers presenting with increased arteriole stiffness perform worse on memory tasks compared to non-carriers [149]. Overall, *APOE4* is associated with reduced blood flow, increased arteriole stiffness, and enhanced sensitivity to hypertension [150]. As such, long-term treatment with angiotensin receptor blockers has been shown to improve memory and neuroinflammation independently of Aβ pathology in female mice expressing human *APOE4* [151]. Similarly, research suggests that vascular endothelial growth factor A (VEGFA) is protective against AD in apoE4-TR mice and could be a promising therapeutic target [152]. In addition, systemic treatment with epidermal growth factor (EGF) improved memory performance of apoE4-TR mice independent of Aβ pathology [153]. Neuropilin 1, a regulator of angiogenesis, also modifies the risk for poor cognitive outcomes based on *APOE4* status [154]. Together these data provide evidence that apoE contributes to vascular pathology in an isoform-dependent manner and could be a viable target for therapies toward AD and related vascular dementias.

Emerging evidence suggests that the breakdown of the BBB may be one of the earliest pathogenic events of cognitive decline and AD pathology. ApoE has been shown to modulate cerebrovascular tight junction integrity independent of CAA in AD brains [155]. Additionally, a study showed that BBB breakdown contributes to cognitive decline in *APOE4* carriers independent of Aβ or tau pathology [156]. A major component of the BBB are vascular endothelial cells, which form tight junctions to create a functional barrier [157]. Interestingly, apoE2 and apoE3 are shown to induce proliferation of vascular endothelial cells while apoE4 decreases their proliferation [158], potentially contributing to the breakdown of the BBB. Studies also indicate that apoE4 disrupts the endothelial BBB integrity by influencing extracellular matrix, cell adhesion machinery, cytoskeleton stability, and translation in brain endothelium. The progressive BBB breakdown in the presence of apoE4 leads to synaptic dysfunction and behavior deficits [159, 160]. BBB breakdown is also shown to be a result of dysfunctional pericytes, which regulate the BBB by controlling gene expression in endothelial cells and inducing polarization of astrocytic end-feet [161]. Pericytes expressing apoE4 have a reduced capacity for supporting endothelial cell function, which leads to impaired BBB integrity and increased susceptibility to cognitive decline [66]. Importantly, accelerated BBB breakdown and degeneration of capillary pericytes can also be recapitulated in amyloid mouse models and human iPSC models [67, 162]. Using a novel iPSC-based 3-D model that recapitulates human BBB in vitro, a recent study showed that dysregulation of calcineurin/NFAT-signaling in pericytes induces apoE4-associated CAA pathology, whereas inhibition of calcineurin reduces apoE expression and vascular amyloid accumulation [67]. Additionally, apoE4 accelerates the degradation of the BBB through activation of the cyclophilin A-matrix metalloproteinase-9 (MMP9) pathway in pericytes, whereas suppression of this pathway improves BBB integrity and prevent apoE4-mediated behavioral deficits in amyloid mouse models [162]. These studies demonstrate that apoE plays a major role in maintaining BBB integrity and that targeting these pathways may present novel therapeutics for AD-related neurodegeneration.

### ApoE and other comorbidities

Many AD patients present with comorbidities including cardiovascular disease (CVD) and type 2 diabetes mellitus (T2DM). Not surprisingly, diabetes appears to accelerate cognitive decline and increase vascular pathology [163]. Interestingly, *APOE4* has been shown to increase the risk for both CVD (OR = 3.0, p = 0.018) and T2DM (OR = 2.2, p = 0.04) independently of AD pathology [164, 165]. In addition, *APOE4* is shown to increase the prevalence and hazard for metabolic syndrome in a cross-sectional study of 4,408 middle-aged men [166]. Evidence suggests that these metabolic deficits can occur before AD pathology becomes evident [167]. Thus, some have proposed research to focus on metabolic dysregulation for novel biomarkers and therapeutic targets for AD pathology. Given the influence of metabolism on modifying AD pathology, Polis and Samson have suggested a new perspective of AD as a complex metabolic disorder, which may offer alternative therapeutic strategies in the future [168].

## Studies that inform therapeutic strategies

While *APOE* has been known to be the strongest genetic risk factor for AD for decades, there are currently no apoE-targeted FDA-approved therapeutics for the treatment of AD. Although apoE has been extensively studied in the context of AD pathogenesis, there is still much more to learn about the mechanisms involved. Recently, a novel “ApoE Cascade Hypothesis” was proposed suggesting that the biochemical and biophysical properties of apoE impact a cascade of events at the cellular and systems levels, which ultimately contribute to the aging-related cognitive decline, pathogenic conditions, and AD disease development [169]. Here, we highlight some of the most recent advances in the field that may help guide novel apoE-targeted therapeutic strategies.

### ApoE in omics analysis

Recent advances in technology have allowed for the generation of bioinformatic data from Omics experiments in iPSCs and mouse models, as well as in AD patients. Collectively, these data have shed light on important genetic targets and pathways that are modulated by *APOE* genotype (Table 1). Recently, RNA-sequencing of isogenic iPSC-derived neurons revealed that apoE4 disrupts pathways related to synaptic formation, which leads to increased synaptic transmission [62]. Additionally, apoE expression is reduced in apoE4 astrocytes compared to apoE3, and the presence of apoE4 was shown to alter cholesterol metabolism and impair the uptake of Aβ [62]. Likewise, apoE4 expression in microglia is associated with less efficient Aβ clearance and activation of inflammatory genes. These data suggest that *APOE4* causes global gene expression changes that may alter cellular function and lead to AD pathology [62]. Similar results can be seen from single nucleus RNA-sequencing of post-mortem human samples, providing further evidence that *APOE* expression plays a major role in AD pathology [170]. In support of these results, transcriptome profiling of microglia during disease progression reveals that apoE may be a key upstream regulator of the transition from homeostatic to DAM [116]. Additionally, transcriptomic analysis of apoE-TR mice found differential regulation of genes involved in energy metabolism in apoE4 mice compared to apoE3 mice, suggesting that apoE4 mice are more vulnerable to bioenergetic deficits which could also induce or exacerbate AD pathology [171]. Transcriptomics analysis of cerebral organoids generated using iPSCs from AD patients reveals genes associated with disrupted RNA metabolism with stress granule formation especially in the presence of *APOE4*. Generating cerebral organoids from isogenic iPSC lines where apoE4 is converted to apoE3 attenuates the apoE4-related phenotypes [172]. This is also recapitulated in a study using apoE-TR mice which identified distinct serum metabolite profiles and upregulated lipid levels in *APOE2* mice compared to *APOE3* and *APOE4* [173]. Furthermore, proteomic analysis of human brain tissue and CSF has revealed apoE isoform-dependent changes, with a reduction in synaptic and mitochondrial function and increased abundance of neuroimmune signaling [174–176]. These data demonstrate the importance of apoE in the initiation and progression of AD pathology across multiple levels. One question the field has been hoping to address with recent bioinformatic approaches is how various genetic risk factors, such as *APOE* and *TREM2*, play a synergistic role and interact through common pathways. Recently, a multi-omics comparison of human ESC-derived microglia-like cell lines found that upregulation of apoE is a converging pathogenic node between *SORL1* and *TREM2* mutant models of AD. These data suggest that various risk factors for AD may have overlapping mechanisms through the upregulation of apoE, specifically in microglia [177].

**Table 1:** APOE isoform effects in aging and AD: Insights from multi-omics and biomarker studies

Cohort	Findings	Source	DOI
*Transcriptomics*			
Normal; AD	• *APOE2/3* AD brains are enriched in complement pathway genes (*C4A*, *C4B*, and *HSPA2)* whose expressions are associated with an increase of pTau231/tTau [296]	Brain	10.1038/s41380-021-01266-z
AD	• *APOE2* is associated with genes involved in protein synthesis, folding and degradation, response to unfolded protein, autophagy, and mitochondrial function [139]	Brain	10.1186/s13195-019-0558-0
Normal; AD	• *APOE* is one of the DAM-like signature genes that is significantly up-regulated in human AD brain assessed by single cell- or single nuclei-RNA Sequencing [64, 297–299]	Brain	10.1038/s41467-020-19737-2 10.1038/s41586-019-1195-2 10.1007/s00401-020-02200-3 10.1007/s00401-021-02263-w
Normal *APOE* mice	• *APOE4* reduces energy expenditure in young females and decreases glucose oxidation by redirecting flux through aerobic glycolysis [300]	Brain Plasma	10.1186/s13024-021-00483-y
*APOE* mice Amyloid mouse models	• *APOE4* increases the expression of *Serpina3* family genes, whereas *APOE2* drives distinct blood metabolome profile [173] • DAM/MGnD/ARM exhibit conserved transcriptional signatures across different AD mouse models with *Apoe* being one of the central regulators [116, 118, 301]	Brain Plasma	10.1016/j.neuron.2020.02.034 10.1016/j.cell.2017.05.018 10.1016/j.immuni.2017.08.008 10.1016/j.celrep.2019.03.099
Normal	• Microglial gene expression modules associated with *APOE4* and sex are also enriched with genes involved in cholesterol absorption and lipid digestion [302] • Microglial gene expression modules associated with *APOE4* and age suggest perturbations in lipid and carbohydrate metabolism as well as microglial activation [302]	Brain	10.1111/acel.13606
Normal; AD *APOE* mice	• *APOE4* astrocytes and microglia demonstrate dysregulated lipid metabolism [179] • *APOE4* alters the matrisome, ECM, and immune pathways in hiPSC-mixed cortical cultures and AD brains [179]	Brain hiPSC	10.1016/j.cell.2022.05.017
*Proteomics*			
Normal; AD	• AIF1, APP, GDI2, HSP90AA1, METAP2, NACA, NCK1, PRDX1, RPS27A, SFTPD and UFC1 are downregulated in AD versus control among APOE4 carriers. [303]	Plasma, brain	10.18632/aging.202950
Normal	• *APOE* genotype is associated with levels of PSD95 in superior temporal cortex in AD (E4/* > E3/E3 > E2/*) [59] • *APOE2* is associated with significantly increased levels of PSD95 in superior temporal cortex [59]	Brain	10.1016/j.neurobiolaging.2005.04.008
Control; AsymAD; AD	• The matrisome module (i.e., extracellular matrix associated proteins) is influenced by the *APOE4* but is not associated with cognitive decline rate after adjustment for neuropathology [304].	Brain	10.1038/s41593-021-00999-y
*Lipidomics*			
Normal; AD	• *APOE4* copy number is positively associated with LysoPE and acylcarnitine species [305] • *APOE4* copy number is negatively associated with PE(O), PE(P), ceramides, and triglycerides versus *APOE2* carriers [305]	Plasma	10.3233/jad-191304
Normal; Aging	• LDL cholesterol levels are genotype dependent (E4/E4 > E4/E3 > E3/E3 > E2/E3 > E2/E2) [306] • *APOE2* is associated with increased levels of most phospholipids (i.e., lysophosphatidylcholines and all PE subclasses) [306] • *APOE4* is associated with reduced levels of phosphatidylinositol relative to *APOE2* and *APOE3* carriers [306]	Plasma	10.3233/jad-190524
AD	• *APOE4* is associated with reduced levels of CAR, LPC, LPE, PA, PC, PE, PI, PS, SM, and ST [139]	Brain	10.1186/s13195-019-0558-0
*Apoe*-KO mice	• Both *APOE3* and *APOE4* treatment reduces hyperlipidemia in a dose-dependent manner, lowering both plasma cholesterol and lipoprotein levels [307] • Expression of *APOE4* increases VLDL-cholesterol and reduces HDL-cholesterol levels relative to apoE3 [307]	Plasma	10.1161/atvbaha.112.301193
*Metabolomics*			
AD	• *APOE4* is associated with reduced LysoPC(18:1), LysoPC(P-18:0), and Cardiolipin [308]	Plasma	10.1016/j.jpba.2019.113088
Normal; Young	• *APOE4* carriers show higher levels of cholesterol relative to *APOE2* carriers [309] • *APOE* influences GlycA, isoleucine, LDL-TG, VLDL-TG, and M-VLDL (E2 < E3 < E4) [309] • *APOE* influences LDL particle diameter (E2 > E3 > E4) [309]	Serum	10.1038/s41598-018-36450-9
*Biomarkers*			
Normal	• *APOE4* is associated with increased LDL, IGF-1, SHBG, bilirubin, triphosphate, ApoB, and total cholesterol, and reduced HDL, HbA1C, lipoprotein A, CRP, GGT, vitamin D, creatine, urate, and urea compared to *APOE3* [310] • *APOE2* is associated with increased HDL, CRP, vitamin D, CysC, ApoA, creatinine, and alkaline phosphatase; and reduced LDL, IGF-1, bilirubin, and ApoB, compared to *APOE3* [310]	Serum blood	10.3233/jad-200338
Normal; Aging	• *APOE2* reduces total cholesterol, LDL, lipoprotein A, and ApoB and increases apoA1 compared to *APOE3* [311]	blood	10.18632/aging.103405
Normal	• *APOE4* is associated with increased SNAP-25 in cognitively normal patients [312]	CSF	10.1016/j.neurobiolaging.2021.02.008
Normal; Aging	• *APOE2* carriers without dementia have reduced Aβ burden, with no differences in tau accumulation [313] • *APOE4* carriers without dementia have increased Aβ burden and tau burden [313]	PET	10.1007/s00259-021-05192-8
AD	• *APOE4* carriers with preclinical AD have increased VILIP-1 [314]	CSF	10.2147/ndt.s235395
AD	• *APOE4* is associated with increased levels of CDH6 and HAGH in AD patients [315]	Plasma	10.1038/s41598-020-65038-5
AD	• Levels of CRP are influenced by *APOE (*E2/E3 > E3/E3 > E3/E4 > E4/E4 > E2/E4) [316] • Levels of ApoB are influenced by *APOE* (E2/E3 < E2/E4 < E3/E3 < E3/E4 < E4/E4) [316] • Levels of IL-13 are influenced by *APOE* (E2/E3 < E2/E4 < E3/E3 < E3/E4 < E4/E4) [316] • Levels of CXCL9 are influenced by *APOE* (E3/E3 > E3/E4 > E4/E4) [316]	Plasma	10.1001/archneurol.2012.1070
*APOE* mice	• *APOE4* is associated with increased NP1 levels [317]	Plasma	10.1016/j.nbd.2018.02.014

***Abbreviations***: *pTau: phosphorylated Tau, tTau: total Tau,* *DAM: disease-associated microglia, MGnD: microglial neurodegenerative phenotype, ARM: activated response microglia, AIF1: Allograft inflammatory factor 1, APP: Amyloid precursor protein, GDI2: Guanosine Diphosphate Dissociation Inhibitor 2, HSP90AA1: Heat Shock Protein 90 Alpha Family Class A Member 1, METAP2: Methionyl Aminopeptidase 2, NACA: Nascent Polypeptide Associated Complex Subunit Alpha, NCK1: Non-catalytic region of tyrosine kinase adaptor protein 1, PRDX1: Peroxiredoxin 1, RPS27A: Ribosomal Protein S27a, SFTPD: Surfactant Protein D, UFC1: Ubiquitin-Fold Modifier Conjugating Enzyme 1, PSD95: Postsynaptic density protein 95, AsymAD: asymptomatic AD, LysoPE: Lysophosphatidylethanolamine, PE: Phosphatidylethanolamine, LDL: Low-density lipoprotein, CAR: Carnitine, LPC: Lysophosphatidylcholine, LPE: Lysophosphatidylethanolamine, PA: Phosphatidic acid, PC: Phosphatidylcholine, PI: Phosphatidylinositol, PS: Phosphatidylserine, SM: Sphingomyelin, ST: Sterol, VLDL: Very low-density lipoprotein, HDL: High-density lipoprotein, LysoPC: Lysophosphatidylcholine, GlycA: Glycoprotein acetylation, TG: Triglyceride, IGF-1: Insulin-like growth factor-1, SHBG: Sex hormone binding globulin, HbA1C: Hemoglobin A1C, CRP: C-reactive protein, GGT: Gamma-glutamyl transferase, CysC: Cystatin C, ApoA: Apolipoprotein A, ApoB: Apolipoprotein B, VILIP-1: Visinin-like protein 1, CDH6: Cadherin 6, HAGH: Hydroxyacylglutathione Hydrolase, IL-13: Interleukin-13, CXCL9: Chemokine ligand 9, NP1: Neuronal pentraxin 1*

Collectively, these studies suggest that not only does *APOE4* increase the risk of developing various non-amyloid neuropathologies, but this risk may be synergistically heightened by altered immunomodulation and inhibited ability to respond to neuronal injuries on a synaptic level to maintain proper signaling networks. Future research on the complex interplay between apoE, various neuropathologies, immunomodulation, and synaptic function will help to shed light on the pathogenic mechanisms of AD and other neurodegenerative diseases. Although much of the therapeutic research in LOAD has been geared toward targeting Aβ and tau pathologies, the role of apoE as a common mediator of several amyloid-dependent and amyloid-independent pathways suggests that apoE itself could be a powerful target upstream of multiple AD pathologies.

### Cell-type specific functions

Studies using iPSC-derived cellular models, animal models, and human brains have revealed cell-type and isoform specific functions of apoE. Using iPSC-derived isogenic lines for *APOE*, it was shown that *APOE4* is associated with altered transcriptomic profiles related to synaptic function in neurons compared to *APOE3*. In astrocytes, apoE4 expression results in intracellular cholesterol accumulation, as well as impairment of Aβ clearance [62]. Another study also showed that apoE4 is associated with impaired lipid and fatty acid metabolism by disrupting neuron-astrocyte coupling. Fatty acid accumulation in neurons leads to disruption in fatty acid oxidation and lipid dysregulation in astrocytes. Compromised lipid metabolism in astrocytes can then affect several downstream pathways, including Aβ clearance [178]. Astrocytic apoE expression has also been shown to affect Aβ clearance and deposition in vivo [85]. In addition, microglia-like iPSC-derived cells expressing *APOE4* exhibit transcriptomic changes associated with immune response, resulting in morphological alterations that correlate with diminished phagocytosis of Aβ, amongst several other downstream effects [62]. Whether apoE isoforms differentially modulate lipid metabolism and neuroinflammation in a cell type-dependent manner warrants further investigation. Interestingly, a very recent study showed that *APOE4* drives lipid metabolic dysregulation in astrocytes and microglia that may contribute to increased AD risk [179]. Together, these data demonstrate that restoring apoE4-mediated dysregulation of Aβ and lipid metabolism in astrocytes and microglia could be a cell-type driven therapeutic avenue.

A genotype-dependent role for *APOE* has also been reported in VMCs, including pericytes. VMC-expressed apoE has been shown to differentially regulate neurobehaviors, gliovascular functions, and transcriptomic profiling depending on isoforms [146]. Transcriptomic profiles in human pericytes isolated from the prefrontal cortex and the hippocampus show an upregulation of apoE4 both at the transcript and at the protein levels compared to apoE3 [67]. Similarly, in a human iPSC-derived BBB model, apoE4-expressing VMCs with pericyte-like properties display elevated apoE levels compared to those expressing apoE3, which could be in part responsible for amyloid accumulation in the vasculature. Thus, *APOE* is differentially regulated depending on the cell-type it is expressed by, supporting the consideration of cell-type specific targeting of apoE [67].

A study using single-nucleus RNA-sequencing of 48 prefrontal cortex tissues brought to light several new findings relating to both sex-dependent and cell type-dependent mechanisms in AD pathophysiology. Cell-type specific changes mostly occurred in the early stages of AD pathogenesis, with disease-related cell population changes occurring predominantly in females. This study also identified disease-driven changes in myelination-related pathways, pointing to a major role for myelination in AD [64]. Understanding how apoE isoforms modulate myelination-related pathways and transcriptomic changes in different cell types during disease progression may further shed light on the pathogenic mechanisms of AD.

### Rare apoE variants associated with AD risk

The *APOE3*-Christchurch (*APOE3*-Ch) variant has sparked interest in the AD field since its homozygosity was linked to remarkable resistance against an aggressive form of familial AD driven by the *PSEN1*-E280A mutation in a single case report [15]. Carrying two copies of this rare allele significantly reduced tau pathology and neurodegeneration in the patient, with preserved glucose metabolism and cognition. The effect on amyloid pathology is unclear since high amyloid burden was still detected by amyloid PET [15]. *APOE3*-Ch was discovered in 1987 as a susceptibility factor for type III hyperlipoproteinemia in Caucasian populations [180]. It encodes a missense mutation on an *APOE3* background which results in an Arg to Ser substitution at position 136, within the receptor binding region of apoE [180, 181] (Fig. 1). In vitro studies using bacterially produced apoE3-Ch have suggested that the R136S mutation leads to reduced binding to the LDL receptor and heparin [15, 181]. How these differences in biochemical properties result in protection against AD is still unclear. It is possible that deficient interaction between apoE3-Ch and HSPG may directly or indirectly impact AD pathologies in a positive manner.

Other rare *APOE* variants have since been linked to either AD risk or AD protection. The *APOE3*-Jacksonville (*APOE3-*Jac) mutation on an *APOE3* backbone results in a Val to Glu substitution at position 236 within the C-terminal domain (Fig. 1). *APOE3*-Jac was linked to reduced risk for AD and dementia with Lewy bodies (DLB) [17, 18]. A recent study showed that apoE3-Jac promotes healthy brain aging and decreases amyloid deposition and associated toxicity by reducing apoE self-association and increasing lipidation [17]. The C-terminal region of apoE is involved in its oligomerization in solution and it also contains a lipid binding region. Given that lipidation of apoE is favored when the protein is in its monomeric form [84], it is possible that the reduced hydrophobicity and/or conformational change in apoE3 caused by the V236E substitution results in its reduced oligomerization and increased lipidation capacity, contributing to its protective role in AD. Other mutations in *APOE*, such as L28P in an *APOE4* backbone (*APOE4*-Freiburg) or *APOE3*-R145C (*APOE3-*Philladelphia) (Fig. 1) have been linked to increase risk for lipid disorders and cardiovascular diseases [182, 183]. However, genetic association of these rare variants to LOAD have not been robustly studied yet [18]. In a more recent report, a new *APOE* protective variant (*APOE4*-R251G) was discovered upon analyses of multiple disease cohorts [19]. Interestingly, this mutation is on an *APOE4* backbone with a Arg to Gly substitution at position 251 within the C-terminal domain of apoE. How this *APOE4*-R251G variant protects against AD risk is not clear although changes in apoE structure and/or lipidation capability are among the possibilities. The location of the protective *APOE3-*Jac and *APOE4*-R251G variants within the carboxyl-terminal portion of apoE suggests that this region of apoE plays an important role in apoE biology and pathobiology. In summary, understanding how mutations in apoE affect its structure, biochemical properties, and function, and how this may translate in differential effects on AD pathobiology, would allow for the identification of new therapeutic avenues and personalized medicine.

## ApoE-Targeted therapeutic strategies

Removal of amyloid plaques is a promising therapeutic avenue in AD and efforts have led to the development of aducanumab, a newly FDA-approved treatment that targets amyloid aggregates [184]. A recent encouraging clinical trial result reported that another anti-Aβ drug, lecanemab, could potentially slow the cognitive decline in people with early onset of AD by 27% over 18 months. However, the long-term safety and effectiveness of this new therapy still need to be explored. Whether *APOE* genotype further modifies the safety and efficacy of this drug will also require further investigation. It is well-known that anti-amyloid immunotherapy can increase the incidence of amyloid-related imaging abnormalities (ARIA) with brain edema or hemorrhage [185]. Interestingly, *APOE4* is greatly associated with ARIA and exhibits a gene dose effect; thus, obtaining *APOE* genotype status has been recommended to be a prerequisite for an AD therapy to better inform ARIA risk, treatment plan, and clinician vigilance [186, 187]. In addition, since *APOE* genotype has been shown as a key determinant of AD risk impacting multiple disease pathways, apoE-targeted therapy has become an attractive avenue for consideration of novel therapies. Here, we outline and discuss the major apoE-related properties and mechanisms being examined as potential apoE-targeted therapeutic strategies.

### Modulating the levels of apoE

Characterization of apoE isoforms in animal models and clinical studies has shown different protein levels among them [188, 189], which appears to be independent of Aβ levels or AD diagnosis [190]. Since apoE plays a major role in Aβ plaque formation [71, 85], several strategies have focused on modulation of apoE levels to prevent or reduce pathological development of AD. ApoE haploinsufficiency was shown to reduce amyloid deposition in amyloid model mice [191]. Furthermore, astrocyte-specific deletion of the *Apoe* gene improves cognitive performance [192] and reduces Aβ deposition and apoE accumulation in the brain of AD mouse models [105]. Thus, the dependence of Aβ on apoE levels has given rise to several strategies aimed toward altering apoE levels for prophylactic and therapeutic benefits.

**Fig. 2:**
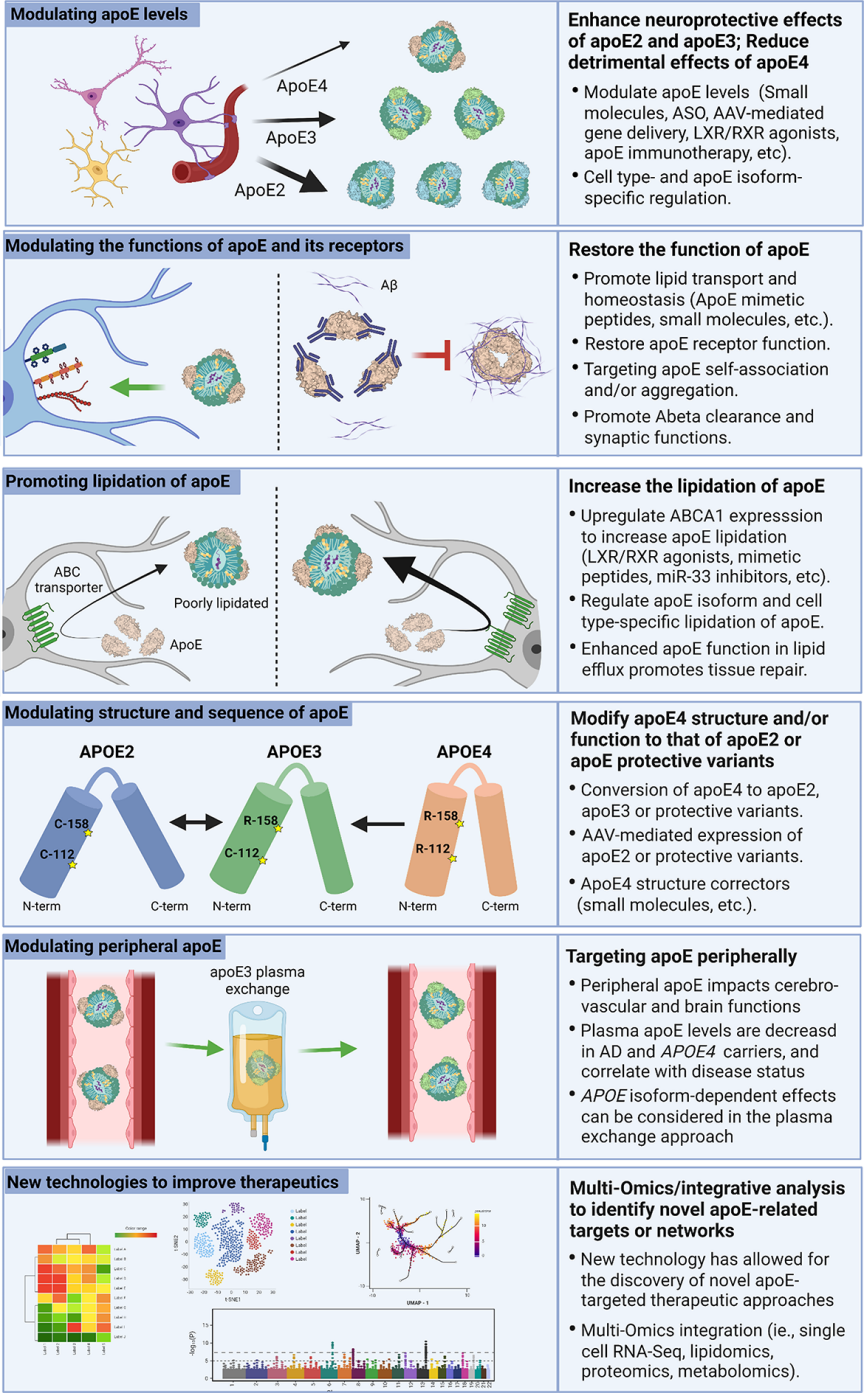
ApoE-targeted therapeutic strategies for AD. One avenue of AD therapy is modulating apoE expression from various cell types. This can be achieved through LXR/RXR agonists which increase apoE levels and lipidation. LXR/RXRs are upstream regulators of apoE expression making them a suitable target for modulating apoE levels. Targeting apoE should also consider the isoform- and cell type-specific effects. Another apoE-targeted therapeutic strategy is the use of small molecules that modulate apoE functions. These include peptides designed to mimic the binding site for apoE on LDL and HDL, which has been shown to increase apoE lipidation and secretion. Additionally, mimetic peptides can increase the function of apoE receptors to improve cholesterol transport. Small molecules or immunotherapies that prevent apoE self-association and/or aggregation may increase the lipid carrying capacity of apoE and reduce Aβ seeding. Similarly, modulating the lipidation of apoE has become an interesting target. Mimetic peptides can be used to increase the activity of ABCA1, which increases the lipidation of apoE4 and improves cognitive function. This can also be achieved through anti-sense oligonucleotide (ASO) inhibition of miR-33. Another promising therapeutic avenue is structural modification of apoE through genetic manipulation or small molecules. The CRISPR/Cas9 system has the potential to directly convert *APOE4* to *APOE3* or *APOE2*. This may also be achieved through an AAV system to induce apoE2 expression. A similar approach without genetic manipulation would be small molecule inhibitors to reduce interdomain interactions and structurally modify apoE to alter its function. Lastly, targeting peripheral apoE may be an alternative avenue for AD therapy. For example, plasma exchange by infusing *APOE3* young plasma in *APOE4* carriers is currently being tested in clinical trials to determine the beneficial effects of young plasma and the isoform-dependent effects. While these various therapeutic approaches have shown some promise in preclinical and clinical settings, they have yet to make a significant impact on the overall prognosis of AD. Research continues to seek alternative approaches to refine the current therapeutic strategies. Presently, many new technologies are being employed to discover new targets and networks such as transcriptomics, proteomics, lipidomics, and metabolomics. These multi-omics and integrative analysis may help better inform future apoE-related disease modifying therapy for AD.

One promising apoE-focused therapeutic avenue is the use of immunotherapies to reduce apoE, in particular apoE4, and consequently alleviate Aβ deposition (Fig. 2). In vivo experiments showed that intraperitoneal injection of HJ6.3, a monoclonal antibody specific against apoE, is effective in reducing amyloid deposition by modulating microglial responses and inflammatory cytokine levels [193]. Administration of HJ6.3 to amyloid model mice reduces Aβ pathology, improves spatial learning performance, and restores functional connectivity without altering plasma cholesterol levels when administered both prior to and after plaque onset [194]. These data demonstrate that reducing apoE4 level may be beneficial in attenuating AD pathology.

### Peptide-based interventions for apoE

Insights into the structure of apoE and its biochemical interactions have opened the door to several mechanisms by which the function of apoE could be modulated. One such mechanism is to inhibit apoE receptor binding through mimetic peptides, short peptide sequences designed to compete for apoE binding sites and thereby reduce its function [24, 195] (Fig. 2). For example, peptides encompassing the apoE receptor binding region (amino acids 130–150) have been shown to reduce microglial immunoreactivity in vitro [196], and improve survival in mouse models of traumatic brain injury [197, 198] and AD [199]. On the other hand, peptide mimetics have also been used to promote apoE function. In one approach, peptides were designed to mimic HDL to bind apoE, thereby increasing apoE secretion and enhancing lipidation status to negate the impacts on Aβ metabolism [200]. These preclinical data support the idea that mimetic peptides might be a useful strategy for therapeutics targeting of apoE function.

Given the role of apoE in amyloid deposition, inhibiting the interaction between apoE and Aβ peptides is another potential strategy for preventing AD pathogenesis (Fig. 2). Peptides binding to residues 12–28 of apoE have been shown to inhibit this interaction [201] and effectively reduce Aβ pathology and synaptic loss in mouse models of AD [202]. Further upstream in the amyloid processing pathway, peptides that prevent apoE binding to APP have been shown to reduce Aβ and tau pathology, improve cognitive performance, and have no effects on lipid profiles in a mouse model [203]. Thus, preventing amyloid deposition by modulating the function of apoE with mimetic peptides demonstrates a promising therapeutic avenue in preclinical studies.

### ApoE self-association and aggregation

ApoE protein is deposited within Aβ plaques and CAA. ApoE4 is believed to have a higher propensity to oligomerize, thus reducing its lipidation and capacity for Aβ clearance as well as facilitating Aβ aggregation [20, 39, 46]. A previous study showed that apoE4 potently increases amyloid deposition by promoting Aβ seeding [85], suggesting that apoE4 may nucleate Aβ aggregation or plaque formation through its self-aggregating propensity. Importantly, both central [chronic intracerebroventricular (i.c.v.) infusion] and peripheral [intraperitoneal (i.p.) injection] administration of an anti-human apoE antibody (HAE-4), which selectively targets poorly-lipidated, aggregated forms of apoE present in the plaque, has been shown to remove Aβ plaques via a microglial-mediated clearance mechanism [204]. Of note, i.p. injection of HAE-4 antibody had a slightly greater effect on reducing Aβ levels than those with i.c.v. infusion likely due to relatively higher HAE-4 antibody levels remaining in the brain after 6 weeks of treatment in the i.p. injection paradigm. Of note, HAE-4 treatment can reduce CAA and Aβ parenchymal plaques after the onset of plaque deposition without inducing ARIA, the adverse vascular effects, and inhibit Aβ-associated tau seeding and spreading [205, 206]. Furthermore, a recent study showed that expression of apoE3-Jac variant which exhibits a decreased self-association reduces the risk of AD and amyloid deposition [17]. These findings suggest that targeting aggregated pools of apoE and/or apoE self-aggregation might be an ideal therapeutic strategy for AD.

### ApoE lipidation status and lipid metabolism

Given that lipidated apoE is more effective in supporting brain homeostasis and injury repair, as well as mediating Aβ clearance compared to non-lipidated apoE, another therapeutic strategy is to alter the physiological balance between lipidated and non-lipidated apoE (Fig. 2). Peptide mimetics used to upregulate ABCA1 have been shown to increase apoE4 lipidation, and reduce Aβ, tau, and cognitive deficits in a mouse model [207]. Similar results may be accomplished by reducing apoE aggregation to increase its ability to accept lipids [204]. The LXR and retinoid X receptors (RXRs) family are known to promote apoE lipidation and expression [208]. Oral administration of bexarotene, an LXR/RXR agonist and FDA-approved anti-cancer agent, resulted in enhanced clearance of Aβ and improved cognitive performance in animal models [209]. However, Phase 1B studies with *APOE3* individuals showed that bexarotene failed to increase CNS apoE or alter Aβ levels, likely due to poor BBB permeability [210]. A different cohort of AD patients, which included diverse *APOE* genotypes, showed that bexarotene treatment did reduce Aβ deposits in several brain areas. However, there were no signs of cognitive improvements and elevated plasma triglycerides as well as liver toxicity were some of the negative consequences observed in the clinical trial [211]. Although the clinical trials for bexarotene in the treatment of AD showed disappointing results, the studies on LXR/RXR agonists highlighted the importance of considering *APOE* genotype in modulating the response to clinical trials and identifying potential therapeutic targets. In addition, administration of choline, a soluble phospholipid precursor, is shown to restore the defective lipid homeostasis in *APOE4* iPSC-derived astrocytes [133]. These studies suggest that modulating the lipidation status of apoE and/or lipid metabolism in the brain may serve as a therapeutic approach to alleviate the lipid dysregulation associated with apoE4.

### ApoE structural correctors

Due to the amino acid substitutions at positions 112 and 158, the amino- and carboxyl-termini of apoE4 have an interdomain interaction which makes the protein more compact compared to apoE2 and apoE3. It has been suggested that this interdomain interaction contributes to the pathogenic effects of apoE4 [212]. Thus, small molecules have been proposed as a method to target and inhibit interdomain interactions [213], essentially acting as a structural corrector of apoE4 (Fig. 2). Human iPSC studies suggest that phthalazinone derivatives may inhibit these domain interactions and thus ameliorate the toxic effects of apoE4 in neurons [214, 215]. This suggests that structural modification of apoE4 may be an alternative approach to reduce the toxic effects of apoE4. Further research on the structure of apoE and its receptors will yield greater mechanistic insight toward this goal.

### Gene therapies targeting ***APOE***

The CRISPR/Cas9 genome-editing system has been explored as a therapeutic avenue for several genetically inherited disorders and is currently being tested in clinical trials to treat certain types of cancers [216, 217]. One intriguing idea is that CRISPR/Cas9 could be used to convert the *APOE* genotype of *APOE4* carriers to *APOE3* or *APOE2*, thus ameliorating the toxic effects of apoE4 and conferring the protective benefits of other isoforms (Fig. 2). In human-derived iPSCs, CRISPR/Cas9 was effective in altering the genome to produce isogenic lines homozygous for all three major *APOE* alleles [218]. In iPSC-derived neurons, CRISPR/Cas9 correction of *APOE4/4* to an *APOE3/3* genotype reduces susceptibility to cytotoxicity, tau secretion, and tau phosphorylation [101]. Experiments in iPSC-derived organoids show that converting *APOE4* to *APOE3* seems to attenuate Aβ pathology by improving astrocytic and microglial clearance of Aβ [62]. Although there are challenges with using CRISPR/Cas9 in vivo, including neuroanatomical specificity, potential off-target effects, and timing of treatment, its potential as a precise and efficacious therapeutic is unlimited.

A viral delivery approach, such as AAV, is another method that has been used to safely and accurately introduce recombinant genes into a host genome [219] and has proven safe and effective for several genetic disorders [220, 221]. Harnessing the protective effects of apoE2 by using AAVs could negate the toxic effects of apoE4. Experiments in mouse models show that transduction with AAV vectors encoding the three apoE isoforms differentially alters Aβ deposition and clearance in an isoform-dependent manner [222]. Furthermore, viral mediated overexpression of apoE2 in apoE4-TR mice enhances apoE lipidation and associated cholesterol, whereas overexpression of apoE4 has an opposite effect [223]. These results suggest that introducing apoE2 in *APOE4* carriers could be beneficial to treating AD. However, AAV-mediated intracerebroventricular delivery of apoE appears to have no effect on tau burden in models of tauopathy [224]. Although there are certainly hesitations regarding CNS delivery of AAVs in humans, work in non-human primates has proven this method to be safe and effective in promoting widespread expression of apoE2 [225]. Furthermore, a phase 1 clinical trial is currently recruiting patients to evaluate the use of intracisternal administration of AAV-mediated expression of *APOE2* in *APOE4* homozygous patients (NCT03634007) with expected completion by September 2024. The efficacy of treating *APOE4* homozygotes with expression of apoE2 may be dependent on the protective effects of apoE2 being able to compensate for the toxic effects of apoE4 which warrants further investigation.

### MicroRNAs

Micro-RNAs (miRNAs) have emerged as important contributors to several pathogenic processes in AD [226], as well as potential biomarkers for disease progression in LOAD [227]. Research has identified miRNAs that contribute to Aβ-induced neurotoxicity [228], synaptic dysfunction [229], immune reactions [230], and tau pathogenesis [231]. One such example is miRNA-33, which has been identified as a key modulator of ABCA1, apoE levels, and subsequently of Aβ metabolism in AD [232]. Targeting of miRNA-33 via ASO results in increased ABCA1 levels, elevated apoE lipidation, and decreased Aβ levels in mouse models [233]. Not only can miRNAs alter apoE function through a variety of mechanisms, but some miRNAs appear to be expressed differentially based on *APOE* genotype. For instance, miRNA-146a is involved in inhibitory feedback for the brain’s immune responses and is reduced in the brain and plasma of *APOE4* mice, thus exacerbating innate immune responses [234]. Further research on miRNAs related to apoE and AD pathogenesis is needed to reveal insights into this mechanistic relationship and the transcriptional networks involved in AD development and progression.

### Targeting the periphery

In addition to the role of apoE in the CNS, the contributions of peripheral apoE have also become a topic of interest. Although there are indeed separate pools of apoE in the CNS and periphery due to BBB impermeability [24, 53, 235], research suggests that peripheral apoE can influence cognitive function [236]. In *Apoe*-deficient animal models, restoration of peripheral, but not CNS apoE reduced synaptic loss and improved cognitive function [237]. However, it seems that reducing the levels of hepatic apoE in the periphery did not influence Aβ pathology in a mouse model in which apoE is present in the CNS [238]. A recent study showed that liver-expressed apoE4 compromises synaptic plasticity and cognitive behaviors likely by impairing cerebrovascular functions [160]. In addition, exposure of young apoE3 plasma ameliorates aging-related BBB damage and promotes endothelial barrier integrity, shedding light on the therapeutic potential of young plasma based on *APOE* genotype. In humans, these effects are also shown to be isoform-specific, perhaps due to *APOE4* carriers having reduced levels of plasma apoE than *APOE3* or *APOE2* individuals [239]. It has been shown that low plasma apoE levels seen in *APOE4* carriers are associated with increased plasma glucose levels, which negatively impacts cerebral glucose metabolism [240]. In addition, researchers have found differences in peripheral extracellular vesicles (pEVs), which may function to promote crosstalk between the brain and periphery. A study reports that pEVs from *APOE4* carriers have reduced neurotrophic and inflammatory markers, and these changes may predict AD five years before symptom onset [241]. Interestingly, apoE deficiency can lead to accumulation of pyrrolated serum albumin, which has a higher binding affinity for apoE3 compared to apoE2 and apoE4 and increases the immune response [242]. Though the mechanism remains unclear, these data suggest a relationship between peripheral apoE expression and inflammatory signaling that is isoform-dependent.

Thus, interventions harnessing the protective function of peripheral apoE in an isoform-specific manner are appealing options with numerous potential mechanisms of action (Fig. 2). A clinical trial targeting the periphery with promising results is the Alzheimer Management by Albumin Replacement (AMBAR) study, where patients undergo plasmapheresis with albumin replacement [243]. However, this study has not considered the influence of age, sex, or *APOE* genotype [244]. To further investigate the potential benefits of peripheral apoE, clinical trials are currently exploring the effects of introducing plasma from young donors to cognitively impaired and AD patients in an isoform-specific manner (NCT02256306, NCT03887741). While these clinical trials are still in early stages, the safety, tolerability, and feasibility appear promising [245]. Alternative approaches could be used to alter levels of apoE in the periphery via genetic interventions, or with the use of structural modifiers to modulate the function of peripheral apoE in an isoform-specific fashion as discussed in earlier sections.

### Lifestyle changes

#### Exercise

**Fig. 3:**
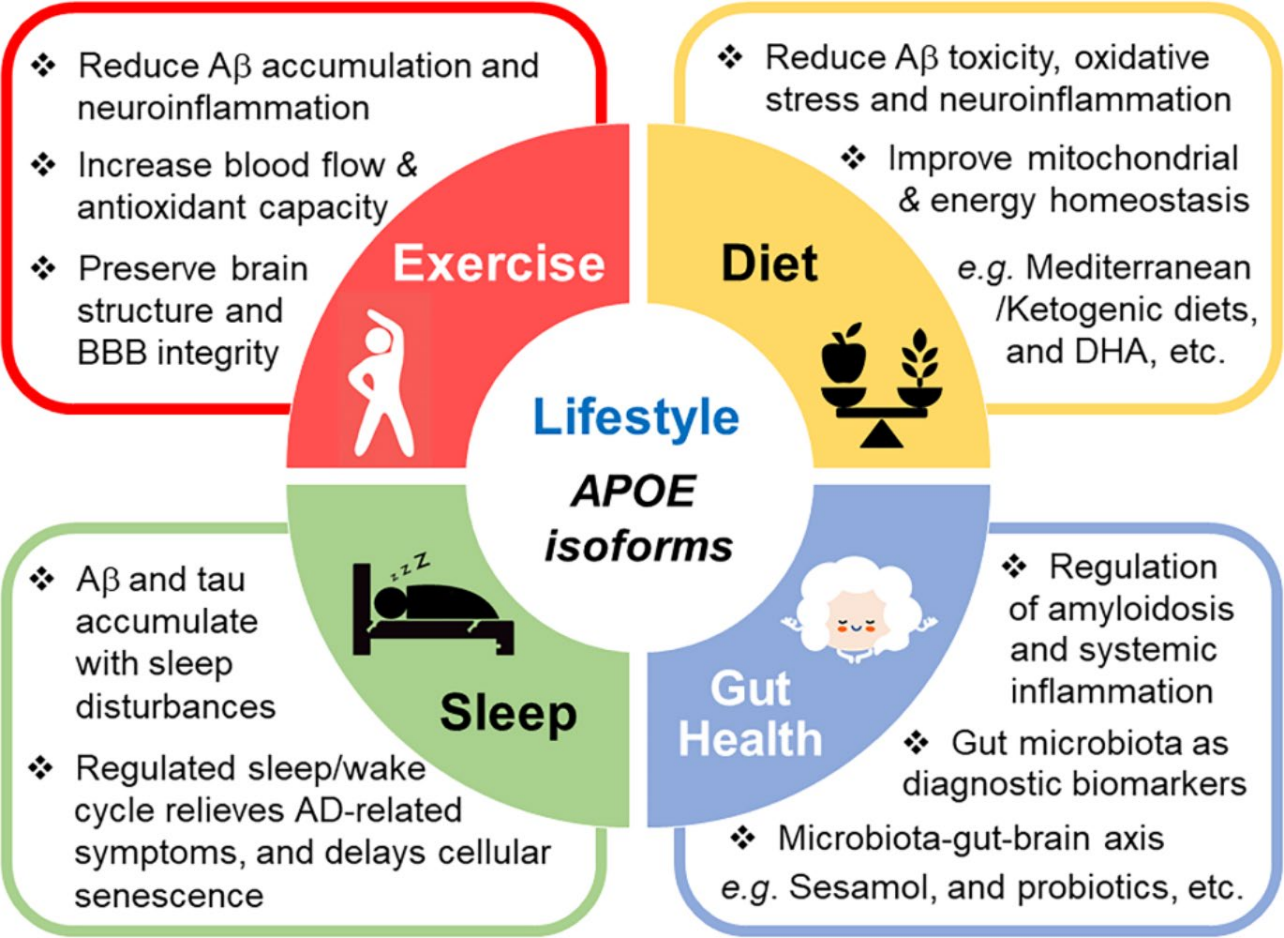
Lifestyle changes can influence the pathogenesis of AD. ApoE4 has been shown to increase Aβ and tau aggregation, inflammation, and lipid dysregulation while reducing glucose metabolism, microbiome diversity, and BBB integrity. Healthy lifestyle changes have been suggested to benefit cognitive function and ameliorate AD pathology even in the presence of *APOE4*. Studies have demonstrated that ketogenic and Mediterranean diets as well as dietary supplements such as DHA can improve clinical outcomes. Along with diet, exercise has been shown to improve AD prognosis in apoE4 carriers. Chronic sleep disturbance appears to accelerate Aβ and tau pathology and exacerbate cognitive symptoms. The influence of *APOE4* on sleep quality may lead to sleep disturbances in people at increased risk for dementia. Thus, improving sleep quality could reduce AD pathology and attenuate the negative impact of *APOE4* on AD risk. The communication between gut microbiome and the brain, the microbiota-gut-brain axis, plays an important role in modulating AD pathology. ApoE isoforms have been shown to differentially modulate microbiome diversity. Evidence supports the use of sesamol to reshape the gut microbiome and prevent systemic inflammation. Thus, understanding the link between AD, apoE, and gut microbiota modulated through dietary approaches may offer avenues for identifying novel biomarkers and therapeutic strategies against AD

Lifestyle changes, such as physical exercise and diets, can potentially attenuate AD pathology. Physical activity has been shown to reduce Aβ accumulation, improve cholesterol levels, reduce neuroinflammation, and enhance cognitive function [246]. Despite some contradictory evidence in the field, it is becoming increasingly apparent that the beneficial effects of exercise and diet are likely dependent on *APOE* genotype [247]. Following the assessment of physical activity and cognitive function in 806 participants over 6 years, a recent study showed that an active lifestyle has a favorable correlation with cognitive performance in *APOE4* non-carriers [248]. Though some studies suggest the effects of the *APOE4* allele are mixed and need to be further characterized [249], many reports provide evidence that *APOE4* carriers are more responsive to exercise interventions compared to non-carriers [250, 251]. Additionally, recent studies have shown that *APOE4* carriers who participated in aerobic exercise show increased hippocampal blood flow, improved verbal memory performance, improved neuropsychiatric symptoms and physical mobility compared to non-carriers and sedentary *APOE4* carriers [252, 253]. Another study shows that physical activity was able to reduce functional connectivity, preserve brain structure, and reduce anxiety levels in *APOE4* individuals [254]. It has been hypothesized that physical exercise and *APOE* genotype impact amyloid clearance and the proteasome system through epigenetic mechanisms [255]. Interestingly, exercise has been shown to increase plasma antioxidant capability and influence Aβ accumulation in *APOE4* carriers [255]. Although the mechanism by which specific exercise paradigms benefit *APOE4* carriers has yet to be established, it remains a valuable therapeutic strategy for reducing or delaying AD symptoms and pathology (Fig. 3).

#### Diet

Preclinical and clinical studies over the past decade have suggested the ketogenic diet to be neuroprotective against AD by reducing Aβ toxicity, decreasing neuroinflammation, protecting against reactive oxygen, and regulating mitochondrial homeostasis [256, 257]. Since apoE is strongly associated with lipid and glucose metabolism, it is no surprise that *APOE* genotype affects preferences for cellular energy sources and modifies the response to diet [258]. The low-carbohydrate, high-fat ketogenic diet provides ketones as energy to mitigate the effects of impaired glucose metabolism in *APOE4* carriers [259]. Recent case studies have shown that 10 weeks of the ketogenic diet was able to improve cognition along with reducing the blood levels of insulin, triglycerides, and glucose in *APOE4* carriers [260, 261]. *APOE4* carriers appear to have a delayed response to ketogenic therapy compared to non-carriers, though the outcomes were still overall positive [262]. A recent review highlights the importance of determining the *APOE*-dependent effects of ketogenic dietary modifications, as well as identifying effective doses of dietary compounds to improve clinical outcomes [263]. Multiple other dietary changes have been proposed to affect AD pathology in an *APOE*-dependent manner including the Mediterranean diet which consists of extra virgin olive oil, capers, red onions, cruciferous vegetables, and fatty fish. These components have been shown to increase LRP1 and ABCA1 levels, decrease NFκB and MMP9 activity, and increase brain docosahexaenoic acid (DHA) levels [264]. In sum, specific dietary changes may provide beneficial outcomes to those at-risk for developing cognitive impairments (Fig. 3).

The potential benefits of dietary supplements, such as DHA have also been extensively studied in the context of AD. It has been shown that *APOE4* carriers have impaired metabolism of plasma DHA, an omega-3 fatty acid partly responsible for BBB integrity, which is associated with behavioral and cognitive impairments [265]. Preclinical and clinical studies of DHA supplementation have had success in rebalancing DHA levels and improving cognitive outcomes [266], although it seems to be ineffective in *APOE4* carriers [267, 268]. Interestingly, it has been proposed that *APOE4* carriers have impaired transport of free DHA into the brain, while increased amounts of DHA in phospholipid form (DHA-lyso-PC) from fish intake has led to positive benefits [269]. This idea has been corroborated by evidence suggesting that disrupted DHA absorption from plasma to CSF exists in *APOE4* carriers and can reduce markers of AD pathology [270]. Further studies will be required to fully elucidate the effects of DHA supplementation on the cognitive function of individuals with different *APOE* genotype.

#### Sleep-wake cycle

Sleep disruptions and nightly restlessness have been a common complaint of AD patients for decades. Disturbed sleep has been shown to result in increased Aβ and tau aggregation, which ultimately leads to sleep disruptions [271]. The potential role of sleep disruption and specific oscillatory patterns are suggested to be useful in diagnosing and tracking the evolution of AD, and as a biomarker for cognitive decline [272]. Mounting evidence suggests sleep disturbances may be worsened or more common in patients who carry *APOE4*, as they have been shown to have reduced rapid eye movement (REM) sleep and increased fragmented sleep compared to non-carriers [273, 274]. Furthermore, *APOE4* exacerbates objective sleep disturbances in individuals, which precedes subjective sleep complaints [275]. This may ultimately lead to longer total sleep duration in *APOE4* carriers with cognitive decline [276, 277]. Importantly, it has been reported that for each sleep interruption, Aβ_42_ clearance is reduced by 5.4%, which is further reduced by the presence of an *APOE4* allele [278]. Thus, understanding the molecular mechanisms by which apoE isoforms influence sleep/awake cycles may facilitate the development of therapeutic approaches to alleviate AD-related sleep disturbances (Fig. 3).

The observance of sleep disturbances in AD patients has led researchers to examine the role of melatonin in sleep regulation for relieving several AD-related symptoms. Melatonin has been shown to reduce Aβ toxicity [279], and reverse the pro-aggregatory and neurotoxic properties of apoE4 [280]. In addition, increased levels of melatonin contribute to cholesterol regulation, choline transport, neurogenesis, tau inhibition, insulin-regulation, calcium homeostasis, mitochondrial function, and can delay cellular senescence [281]. However, compared to age-matched controls, melatonin levels in the CSF are decreased in AD patients, and this reduction is exacerbated by the presence of *APOE4* [282]. Thus, melatonin supplementation has been explored as a remedy for AD-related sleep disturbances. Recently, a meta-analysis of randomized control trials concluded that while melatonin improves total sleep time and sleep efficacy, cognitive function is not significantly altered [283]. Thus, the potential of melatonin for future treatments of AD merits continued research efforts [284, 285]. Recent studies corroborate the need for earlier intervention, and to consider *APOE* genotype when testing interventions targeting the sleep-wake cycle in AD patients [273, 275, 277, 286]. These findings suggest that *APOE* genotype may be one of the critical factors impacting the efficacy of melatonin-based therapies.

#### Gut microbiome

More recent evidence suggests an emerging role for the gut microbiome in modulating AD pathology through metabolism and immune function [287] (Fig. 3). Interestingly, work in transgenic mouse models demonstrated that gut microbiome species are modulated by both *APOE* genotype and gender [288–290]. Furthermore, it has been shown that AD patients have altered gut microbiota, which is associated with *APOE* genotype [291]. Research has also shown ways to modulate the gut microbiome in a beneficial way. In an *APOE4* mouse model, insulin supplementation was shown to increase the levels of beneficial microbiota which increases metabolism and reduces neuroinflammation [292]. Additionally, sesamol has been shown to reshape the gut microbiome and prevent systemic inflammation in an *APOE*-dependent manner [293]. The effects of diet, microbiome, and inflammation on AD pathogenesis further support the notion of a critical communication between the brain and the periphery [294, 295].

Together, understanding the relationship between *APOE* genotype, exercise, diet, and microbiome is necessary for elucidating the underlying mechanisms that drive AD pathology, which may inform the search for robust biomarkers for successful interventions and therapeutics.

## Conclusion

Mounting evidence confirms that apoE plays a key role in the pathogenesis of AD. ApoE4 conveys risk by a combination of gain of toxic functions and loss of protective functions, while apoE2 is protective against AD with overlapping and distinct mechanisms. ApoE impacts amyloid, tau, and additional neuropathologies, while also influencing neurodegeneration and the immune responses to those insults. The role of apoE as an upstream mediator in complex pathways underlying neurodegeneration and cognitive decline makes it an ideal therapeutic target for AD and related dementias. As we gain an enhanced understanding of the structure-function relationship of apoE, as well as the mechanistic relationships between apoE and neuropathologies, we will be able to use therapeutic tools to modulate apoE levels, structure, lipidation, oligomerization, and related outcomes to alter pathological progression. Further studies exploring systematic changes via multi-omics analysis, cell type-specific functions, and novel *APOE* variants may further yield critical insights in the future of AD therapeutic development. In addition, adopting new techniques for research and therapeutic delivery will prove vital in the development of novel treatment options for AD.

## Data Availability

Not applicable.

## Abbreviations

**AD**: Alzheimer’s disease
**Aβ**: Amyloid-β
**APOE**: Apolipoprotein E
**LOAD**: late-onset AD
**NFTs**: neurofibrillary tangles
**eFAD**: early-onset familial AD
**APP**: amyloid precursor protein
**PSEN1**: presenilin 1
**PSEN2**: presenilin 2
**CNS**: central nervous system
**NMR**: nuclear magnetic resonance
**ABC**: ATP-binding cassette
**LDLR**: low-density lipoprotein receptor
**HSPG**: heparan sulfate proteoglycans
**VLDL**: very low-density lipoproteins
**HDL**: high-density lipoproteins
**PET**: positron emission tomography
**ASOs**: antisense oligonucleotides
**iPSC**: induced pluripotent stem cell
**AAV**: adeno-associated virus
**FTD**: Frontotemporal dementia
**DAM**: disease-associated microglia
**MGnD**: microglial neurodegenerative
**TREM2**: triggering receptor expressed on myeloid cells 2
**VCID**: impairment and dementia
**CAA**: cerebrovascular amyloid angiopathy
**DLB**: dementia with Lewy bodies.
